# GCSAM: Gradient Centralized Sharpness Aware Minimization

**DOI:** 10.1109/access.2025.3624332

**Published:** 2025-10-23

**Authors:** MOHAMED HASSAN, ALEKSANDAR VAKANSKI, BOYU ZHANG, MIN XIAN

**Affiliations:** Department of Computer Science, University of Idaho, Idaho Falls, ID 83402, USA

**Keywords:** Deep learning, generalization, gradient centralization, loss landscape, sharpness-aware minimization

## Abstract

The generalization performance of deep neural networks (DNNs) is a critical factor in achieving robust model behavior on unseen data. Recent studies have highlighted the importance of sharpness-based measures in promoting generalization by encouraging convergence to flatter minima. Among these approaches, Sharpness-Aware Minimization (SAM) has emerged as an effective optimization technique for reducing the sharpness of the loss landscape, thereby improving generalization. However, SAM’s computational overhead and sensitivity to noisy gradients limit its scalability and efficiency. To address these challenges, we propose Gradient-Centralized Sharpness-Aware Minimization (GCSAM), which incorporates Gradient Centralization (GC) to stabilize gradients and accelerate convergence. GCSAM normalizes gradients before the ascent step, reducing noise and variance, and improving stability during training. Our evaluations on both general vision benchmarks (CIFAR-10, CIFAR-100) and critical medical imaging datasets (breast ultrasound and COVID-19 chest X-rays) demonstrate that GCSAM consistently outperforms SAM and the Adam optimizer in terms of generalization and computational efficiency. These results highlight GCSAM’s potential for improving reliability in domains where robust generalization is essential, particularly in medical image analysis. Our code is available at https://github.com/mhassann22/GCSAM

## I. INTRODUCTION

Understanding the generalization behavior of overparameterized deep neural networks (DNNs) has recently become a important area of study, as it offers valuable insights into handling overfitting and enhancing performance on unseen data [1], [2]. As the scale of models and datasets continues to expand, the development of optimization algorithms that enhance generalization capabilities becomes increasingly critical. To better understand the generalization behavior of DNNs, comprehensive empirical studies by Jiang et al. [3] and Dziugaite et al. [4] have evaluated various generalization metrics, finding that sharpness-based measures exhibit the strongest correlation with generalization performance. Based on these findings, optimizers that lead to flatter minima, rather than sharp minima, are particularly effective in promoting generalization, as flatter minima have been shown to correlate strongly with improved model performance in overparameterized setting [5], [6].

Sharpness-Aware Minimization (SAM) [7] is a promising optimization technique for finding flatter minima to improve generalization, which achieved consistent improvement in generalization performance across various natural image and language benchmarks [8], [9], [10], [11]. SAM regularizes the sharpness of the loss landscape by simultaneously minimizing the training loss and the loss sharpness. SAM achieves this by employing adversarial perturbations ϵ to maximize the training loss $L_{S}(w+\epsilon )$, where w is the weight vector. It then minimizes the loss of this perturbed objective using an update step from a base optimizer, such as Adam [12]. To compute ϵ, SAM takes a linear approximation of the loss objective, and uses the gradient $\nabla L_{S}(w)$ as the ascent direction for computing $\epsilon =\rho \cdot norm\left(\nabla L_{S}(w)\right)$, where ρ is the radius of the maximization region, and $norm(x)=x/\|x{\|}_{2}$.

However, SAM introduces a significant computational overhead, as the ascent step requires additional forward and backward passes, which doubles the training time. Furthermore, the two-step training process requires tuning additional hyper-parameters, which if not selected correctly, can lead to suboptimal performance or inefficient training. For instance, Wu et al. [13] and Chen et al. [14] demonstrate that the perturbation radius ρ should be adjusted beyond the range initially proposed by Foret et al. [7] to achieve optimal results. Additionally, while SAM successfully regularizes the sharpness of the loss landscape, it does not address the issue of noisy and high-variance gradients, which can hinder the generalization performance.

To address these limitations, we propose Gradient Centralized Sharpness Aware Minimization (GCSAM), which integrates Gradient Centralization (GC) [15] into the ascent step of SAM. GCSAM reduces gradient noise and stabilizes the perturbation direction. This results in more reliable sharpness estimation, smoother optimization trajectories, and faster convergence. References [15], [16], and [17] Moreover, the use of GC makes GCSAM less sensitive to the perturbation radius ρ, improving robustness across different datasets and architectures.

Crucially, the integration of GC into SAM is not merely additive. We show that GC directly addresses a key shortcoming of SAM, the amplification of gradient noise during sharpness-aware ascent, by smoothing the perturbation direction and improving the Lipschitzness of both the loss and its gradient. This leads to smaller, more stable perturbations and tighter generalization bounds, as we formally prove in Appendix A. Unlike prior work such as GSAM, ASAM, or CRSAM, which focus on reparameterizing or regularizing the sharpness objective, GCSAM improves the core optimizer behavior by stabilizing the curvature-sensitive components of SAM itself. In summary, our main contributions are as follows:

- We propose GCSAM, a novel algorithm that combines Gradient Centralization (GC) with Sharpness-Aware Minimization (SAM). This integration targets both sharpness reduction and gradient stabilization, resulting in improved generalization.
- GCSAM addresses limitations in SAM by reducing computational overhead and mitigating issues of gradient noise. By centralizing gradients, GCSAM provides a more stable optimization process that reduces gradient explosion, enhancing both efficiency and robustness.
- We validate GCSAM’s performance on a range of benchmarks, demonstrating improved generalization in both general and medical imaging datasets. Our results indicate GCSAM’s ability to outperform SAM and baseline optimizers in achieving higher test accuracy with enhanced computational efficiency.

## II. RELATED WORK

### A. LOSS SHARPNESS AND GENERALIZATION

The connection between the geometry of the loss landscape and generalization has been the subject of extensive research effort. Chaudhari et al. [18] proposed Entropy-SGD, an approach aimed at minimizing the local entropy of the loss landscape to guide the optimization process toward flatter regions, thereby improving generalization. Similarly, Smith and Le [19] demonstrated that incorporating noise in SGD prevents optimization from entering sharp valleys. Furthermore, Lyu et al. [20] revealed that gradient descent has an inherent bias toward reducing sharpness, especially in the presence of normalization layers [21] and weight decay [22], which further supports the importance of managing the sharpness of the loss landscape to improve generalization in DNNs.

### B. IMPROVING SAM

Several variants of SAM have been proposed to enhance its performance and address its limitations. Adaptive-SAM (ASAM) [8] modifies SAM by introducing a scaling operator that eliminates sensitivity to model parameter rescaling, allowing more robust generalization across different models. Kim et al. [23] refined both SAM and ASAM by leveraging Fisher information geometry, providing a more effective method of minimizing sharpness in the parameter space. Zhuang et al. [9] highlighted that minimizing the perturbed loss in SAM does not always guarantee a flatter loss landscape, leading to the development of Surrogate-Gap SAM (GSAM), which incorporates a measure akin to the dominant eigenvalue of the Hessian matrix to capture sharpness more accurately. Additionally, Wu et al. observed that SAM’s one-step gradient approach may lose effectiveness due to the non-linearity of the loss landscape. To address this, they proposed Curvature Regularized SAM (CR-SAM) [10], which integrates a normalized Hessian trace to more precisely measure and regulate the curvature of the loss landscape. Kaddour et al. introduced Weight-Averaged SAM (WASAM) [24], which integrates SAM with Stochastic Weight Averaging (SWA) [25]. Their findings demonstrate that WASAM enhances SAM’s generalization performance, particularly in Natural Language Processing (NLP) tasks.

In addition to improving performance, several algorithms have been proposed to mitigate the computational overhead associated with SAM. Liu et al. introduced LookSAM [26], which accelerates SAM by periodically computing the inner gradient ascent at every *k*th iteration instead of at each step, reducing the frequency of expensive updates. Du et al. proposed Efficient SAM (ESAM) [27], which minimizes the number of input samples in the second forward and backward passes by utilizing Stochastic Weight Perturbation (SWP) and Sharpness-sensitive Data Selection (SDS) to balance efficiency and sharpness minimization. Furthermore, Becker et al. developed Momentum SAM (MSAM) [28], incorporating Nesterov Accelerated Gradient (NAG) [29] to perturb parameters along the direction of the accumulated momentum vector, which improves both the convergence rate and computational efficiency of SAM.

### C. GRADIENT OPTIMIZATION

Significant research effort has been dedicated to stabilizing and accelerating DNN training through operations on gradients. Gradient clipping [30], [31], [32] was introduced to mitigate the issue of exploding gradients by limiting their magnitude during backpropagation. Qian [33] explored the use of momentum to accelerate gradient descent optimizers and reduce oscillations, enhancing convergence speed. Further, Riemannian methods [34] and projected gradient techniques [35], [36], [37] were employed to regulate weight learning by projecting gradients onto subspaces, promoting more stable learning trajectories. Smith et al. [38] leveraged the gradient norm to derive an implicit regularization term in stochastic gradient descent (SGD), aiding generalization. Additionally, $\ell_{2}$ regularization of weight gradients remains one of the most widely adopted strategies to improve the generalization capabilities of DNNs [22], [39].

## III. METHOD

In this section, we outline the methodological approaches of three techniques: Sharpness-Aware Minimization (SAM), Gradient Centralization (GC), and our proposed Gradient Centralized Sharpness-Aware Minimization (GCSAM).

### A. SHARPNESS AWARE MINIMIZATION (SAM)

While empirical risk minimization algorithms, such as SGD and Adam, effectively reduce the empirical loss $L_{S}(w)$ to achieve low training error, addressing the generalization gap $L_{D}(w)-L_{S}(w)$ remains a challenge in DNN training. Keskar et al. [40] proposed that there is a connection between the sharpness of minimized empirical loss and the generalization gap, as the large sensitivity of the training function at a sharp minimizer negatively impacts the trained model’s ability to generalize on new data. To formalize this connection, sharpness is defined within an ϵ-ball as:

(1) $$\underset{\|\epsilon {\|}_{p}\leq \rho }{max}\;L_{S}(w+\epsilon )-L_{S}(w),$$

where ρ is the radius of maximization region of an $\ell^{p}$ ball. From the above definition, sharpness is the difference between the maximum empirical loss in the $\ell^{p}$ ball and the empirical loss.

Foret et al. [7] introduced Sharpness-Aware Minimization (SAM), which integrates sharpness minimization with a PAC-Bayes norm to improve generalization. SAM is designed to enhance generalization by minimizing the sharpness of the loss landscape, encouraging convergence to flat minima. This is achieved by optimizing the following PAC-Bayesian generalization error bound:

(2) $$L_{D}(w)\leq \underset{\|\epsilon {\|}_{p}\leq \rho }{max}\;L_{S}(w+\epsilon )+h(\frac{\|w{\|}_{2}^{2}}{\rho^{2}}),$$

where the monotonic nature of h allows substitution with an $\ell^{2}$ weight-decay regularizer. Thus, SAM can be formulated as a minimax optimization problem, aiming to mitigate generalization error while maintaining training stability:

(3) $$\underset{w}{min}\;\underset{\|\epsilon {\|}_{p}\leq \rho }{max}\;L_{S}(w+\epsilon )+\frac{\lambda }{2}(\frac{\|w{\|}_{2}^{2}}{\rho^{2}}).$$

### B. GRADIENT CENTRALIZATION (GC)

Gradient Centralization (GC) [15] is an optimization technique that improves training stability and generalization by enforcing zero-mean gradients during backpropagation. Unlike batch normalization and gradient normalization methods [41], [42], which directly modify activation matrices, GC operates directly on gradient vectors or matrices.

Let $g_{t}=\nabla L_{S}\left(w_{t}\right)$ denote the mini-batch gradient at training step t, where n is the number of elements in $g_{t}$. For an individual weight vector $w_{i}$, the centralized gradient is computed by subtracting the mean of its entries:

(4) $$\nabla_{GC}L_{S}\left(w_{i}\right)=\nabla L_{S}\left(w_{i}\right)-\frac{1}{n}\sum_{j=0}^{n-1}\nabla L_{S}\left(w_{ij}\right).$$

**FIGURE 1.**
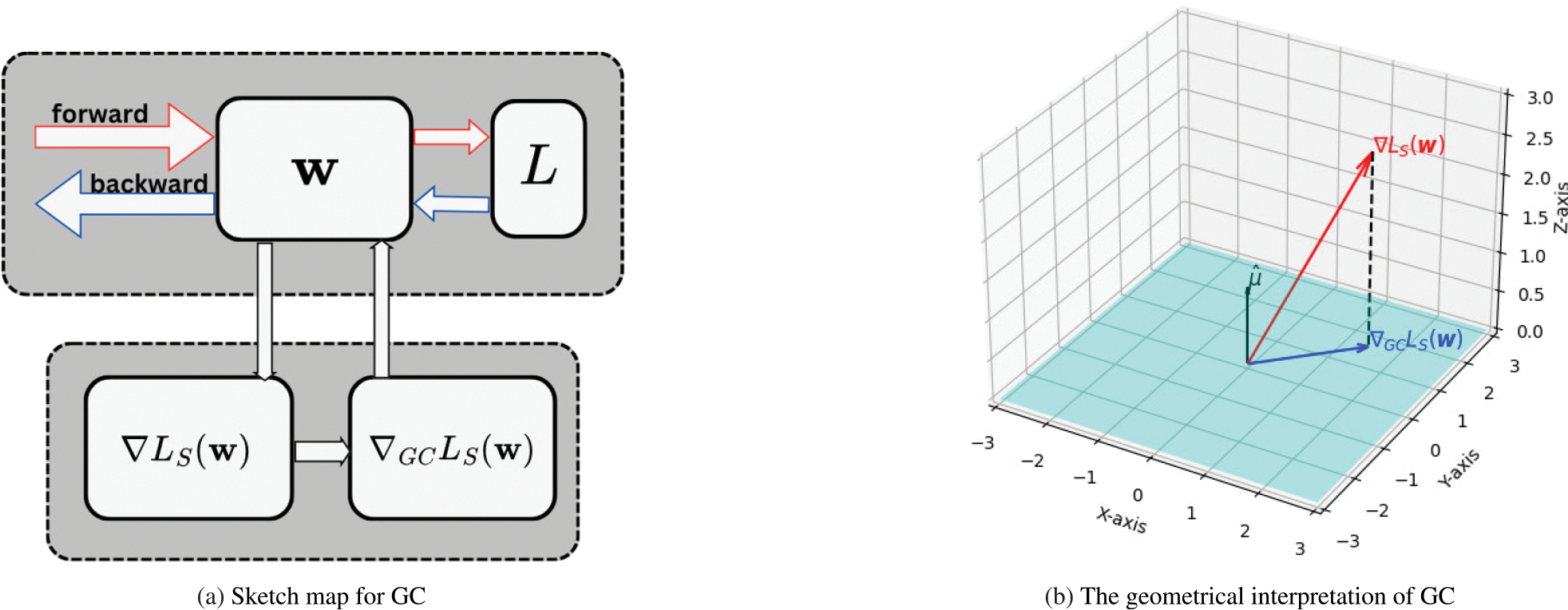
(a) Sketch map for using Gradient Centralization (GC). L is the loss function, w denotes the weight matrix, $\nabla L_{S}(w)$ is the gradient of the entire weight matrix, and $\nabla_{GC}L_{S}(w)$ is the centralized gradient. (b) Geometrical interpretation of GC. The gradient is projected on a hyperplane $\overset{ˆ}{\mu }$, where the projected gradient is used to update the weight.

**FIGURE 2.**
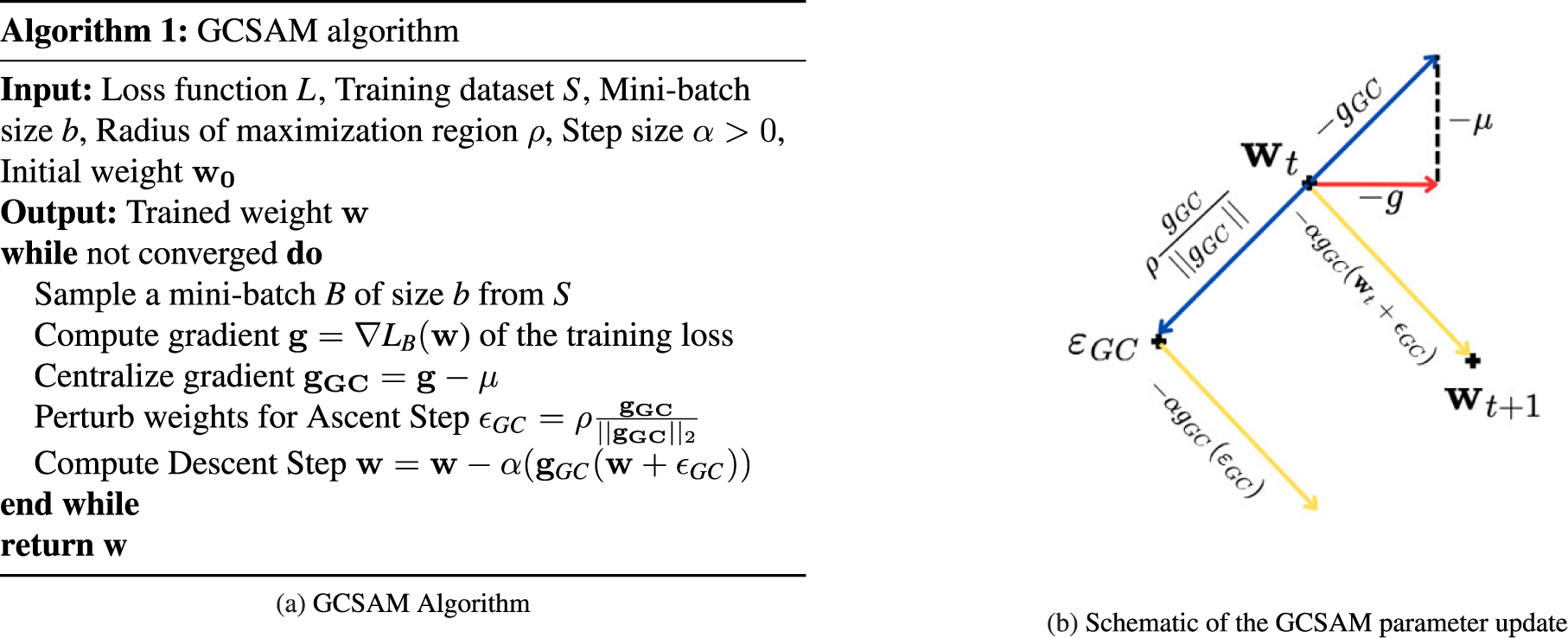
(a) GCSAM Algorithm. (b) Schematic of the GCSAM parameter update, where $w_{t}$ is the current step, $\epsilon_{GC}$ perturbs weights for the ascent step, and $w_{t+1}$ is the next step. (Note: The illustration in (b) is schematic and not drawn to scale. While the centralized gradient appears visually larger for clarity, Eq. (6) formally guarantees that the magnitude of the centralized gradient is always less than or equal to that of the original gradient.).

Equivalently, letting $\mu_{t}=\frac{1}{n}\sum_{i=1}^{n}g_{t,i}$ denote the mean gradient, we can express GC compactly as

(5) $$\nabla_{GC}L_{S}\left(w_{t}\right)=g_{t}-\mu_{t}=Pg_{t},$$

where $P=I-{ee}^{⊤}$ is the projection matrix and e is the allones unit vector. This shows that GC removes the component of $g_{t}$ that lies in the direction of e, constraining updates to a hyperplane orthogonal to the mean direction, as illustrated in Fig. 1b and Fig. 2b.

In practice, GC is applied per parameter tensor and per step, excluding 1-D tensors such as bias and batch-norm parameters to avoid degenerate centralization. The averaging operator in Eq. (4) denotes the per-tensor mean computed from a single mini-batch gradient tensor and subtracted immediately. This per-step operation is conceptually distinct from an exponential moving average (EMA) computed across training iterations. A GC-EMA variant would maintain a running mean

$$\mu_{t}=\beta \mu_{t-1}+(1-\beta )mean\left(g_{t}\right),$$

and subtract $\mu_{t}$ from $g_{t}$ at step t. While this may further smooth temporal fluctuations, it introduces the additional hyperparameter β and interacts nontrivially with optimizer moment estimates (e.g., Adam’s moving averages). For clarity and consistency with prior GC work, we adopt the per-step formulation as default and discuss GC-EMA as a variant in Sec. IV (Ablation Study/GC-EMA variants).

This operation leads to two key benefits. First, GC reduces the magnitude of the gradient, which suppresses gradient explosion and stabilizes optimization. As formally shown in Appendix (A.4):

(6) $${\left\|\nabla_{GC}L_{S}(w)\right\|}_{2}^{2}\leq {\left\|\nabla L_{S}(w)\right\|}_{2}^{2},$$

ensuring that the centralized gradient is always smaller in magnitude than the original gradient. This suppression leads to more stable optimization steps and prevents abrupt parameter shifts during training.

Second, as proven in prior work [15], GC improves the Lipschitz smoothness of both the loss and its gradient:

(7) $$\begin{array}{c} {\left\|\nabla_{GC}L_{S}(w)\right\|}_{2}\;\leq {\left\|\nabla L_{S}\left(w\right)\right\|}_{2},\\ {\left\|\nabla \nabla_{GC}L_{S}(w)\right\|}_{2}\;\leq {\left\|\nabla^{2}L_{S}\left(w\right)\right\|}_{2}, \end{array}$$

where $\nabla^{2}L_{S}(w)$ refers to the Hessian matrix. By improving the Lipschitzness of the loss and its gradients, GC yields a smoother and more predictable optimization trajectory, enabling faster and more reliable convergence toward flatter minima. Moreover, when GC is applied only to the ascent step of SAM, it stabilizes the perturbation direction without altering the descent update, which is empirically shown to produce the best balance of stability and generalization in Sec. IV (Ablation Study/GC Placement).

### C. GRADIENT CENTRALIZED SHARPNESS AWARE MINIMIZATION (GCSAM)

While SAM improves generalization by optimizing for flatter minima, it is often sensitive to noisy gradients, especially in the early stages of training. The sharpness objective amplifies such noise during the inner maximization step, leading to unstable perturbations and inefficient convergence. To address this, we propose Gradient Centralized Sharpness-Aware Minimization (GCSAM), which integrates GC into the sharpness-aware optimization framework. Specifically, GCSAM applies GC to the gradient used in SAM’s ascent step, resulting in a reformulated objective:

(8) $$\underset{w}{min}\;\underset{{\left\|\epsilon_{GC}\right\|}_{p}\leq \rho }{max}\;L_{S}\left(w+\epsilon_{GC}\right)+\frac{\lambda }{2}(\frac{\|w{\|}_{2}^{2}}{\rho^{2}}),$$

where $\epsilon_{GC}$ denotes perturbations aligned with the centralized gradient $\nabla_{GC}L_{S}(w)$:

(9) $$\epsilon_{GC}=\rho \frac{\nabla_{GC}L_{S}(w)}{{\left\|\nabla_{GC}L_{S}(w)\right\|}_{p}},$$

**FIGURE 3.**
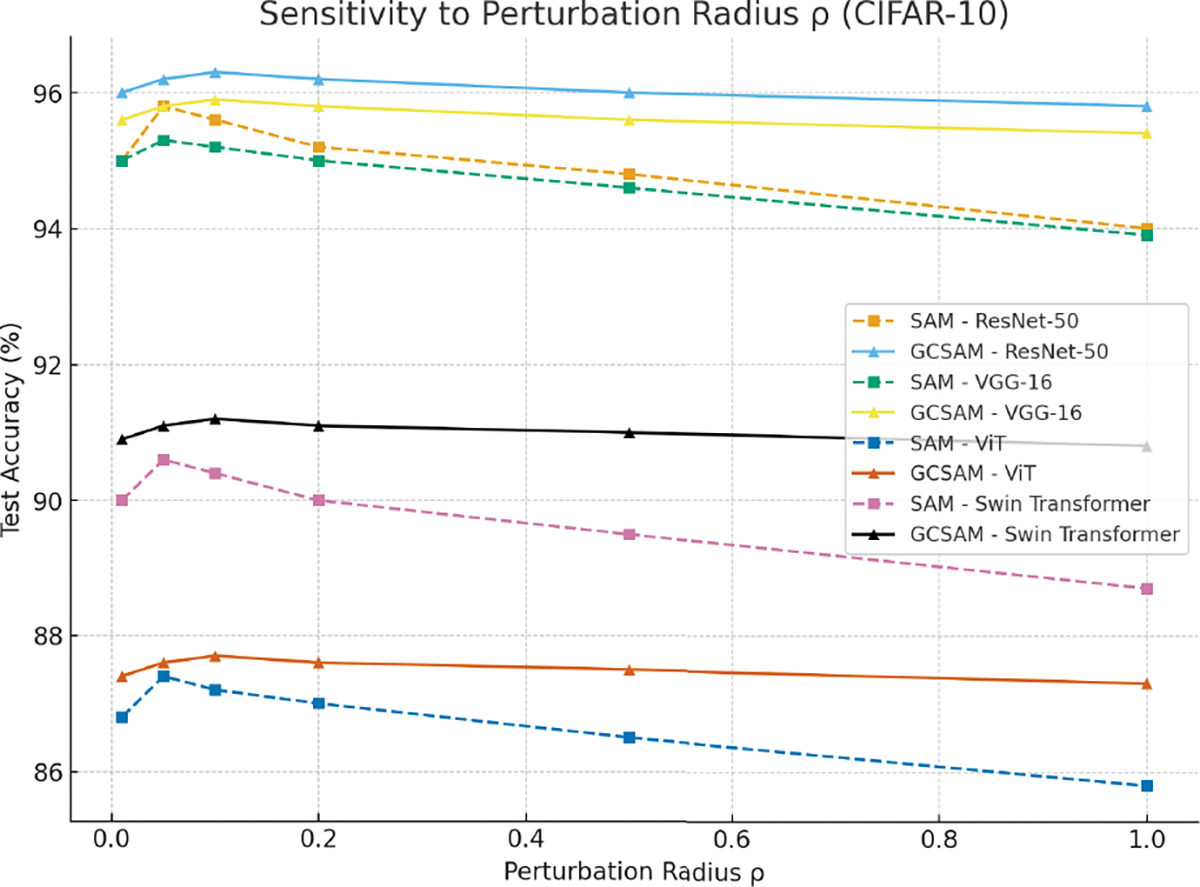
Sensitivity to perturbation radius ρ on CIFAR-10 across four architectures. SAM achieves peak accuracy around ρ=0.05 but exhibits sharp degradation for other values, while GCSAM achieves peak accuracy at ρ=0.1 and maintains stable performance across all tested radii. The lower variance of GCSAM confirms its reduced sensitivity to ρ and greater robustness across architectures.

This centralized perturbation suppresses noise and improves the quality of the sharpness estimation. As a result, GCSAM benefits from tighter sharpness control and more stable updates. Importantly, because gradient centralization reduces gradient variance (Eq. (6)), the perturbation $\epsilon_{GC}$ is less amplified by noisy directions, leading to stable updates even when ρ takes larger or smaller values. This theoretical property explains the empirical robustness of GCSAM to perturbation radius choices, as demonstrated later in Fig. 3.

The final GCSAM algorithm is formulated by integrating a standard numerical optimizer, such as SGD or Adam, as the base optimizer for the GCSAM objective. This combination allows for efficient gradient-based optimization while leveraging the benefits of gradient centralization within the SAM framework. The pseudo-code for the GCSAM algorithm, provided in Fig. 2a, outlines the proposed approach with SGD as the base optimizer, highlighting the procedural steps involved in each update cycle. Additionally, Fig. 2b provides a schematic illustration of a single GCSAM parameter update, visually capturing the interaction between gradient centralization and sharpness-aware minimization in the update process.

To better understand the generalization behavior of GCSAM, we provide a PAC-Bayesian generalization bound that highlights the advantages of applying Gradient Centralization in the SAM framework. By projecting the gradient onto a subspace orthogonal to its mean, GCSAM effectively suppresses sharp gradient directions, leading to a tighter bound on the loss under perturbations.

#### Theorem 1:

Let $\nabla_{GC}L_{S}(w)=P\nabla L_{S}(w)$, where $P=I-{ee}^{T}$ and e is a unit vector. If $L_{D}(w)\leq E_{\epsilon \sim 𝒩\left(0,\sigma^{2}\right)}\left[L_{D}(w+\epsilon )\right]$ for some σ>0, then with probability 1-δ:

(10) $$\begin{array}{l} L_{D}(w)\leq \;\underset{{\left\|\epsilon_{GC}\right\|}_{p}\leq \rho }{max}\;L_{S}\left(w+\epsilon_{GC}\right)\\ \;+\sqrt{\frac{1}{n\;-\;1}\left(k\;log\left(1+\frac{\|w{\|}_{2}^{2}}{\eta^{2}\rho^{2}}{\left(1+\sqrt{\frac{log\;n}{k}}\right)}^{2}\right)+4\;log\frac{n}{\delta }+O(1)\right)} \end{array}$$

where n=|S|, $\rho =\sqrt{k}\sigma (1+\sqrt{log\;n/k})/n$, and $\epsilon_{GC}=\rho \frac{\nabla_{GC}L_{S}(w)}{{\left\|\nabla_{GC}L_{S}(w)\right\|}_{p}}$.

Theoretical analysis in Appendix A further confirms that the GCSAM bound is strictly tighter than the SAM bound due to the reduced centralized gradient norm.

## IV. EXPERIMENTAL RESULTS

To evaluate the effectiveness of GCSAM, we apply it across a diverse set of tasks, including image classification on CIFAR-10 and CIFAR-100, and medical imaging challenges with Breast Ultrasound (BUS) and COVID-19 datasets. In each case, we benchmark GCSAM against multiple baselines: the Adam optimizer, Gradient Centralization (GC) [15] applied to Adam, the original SAM, and recent SAM variants including Adaptive SAM (ASAM) [8], Surrogate-Gap SAM (GSAM) [9], Curvature-Regularized SAM (CRSAM) [10], and Momentum SAM (MSAM) [28]. We evaluate these methods across a variety of architectures, including CNN-based models such as ResNet-50 [43] and VGG-16 [44], as well as Vision Transformer architectures like ViT [45] and Swin Transformer [46]. This experimental setup enables a systematic assessment of GCSAM’s benefits relative to both classical optimizers and advanced sharpness-aware methods in both general-purpose and domain-specific applications.

### A. HYPERPARAMETER TUNING STRATEGY

To ensure fair and unbiased comparisons, all hyperparameters were tuned using a unified grid-search procedure. For sharpness-aware optimizers (SAM, ASAM, GSAM, CRSAM, MSAM, and GCSAM), the perturbation radius ρ was searched over {0.05, 0.1, 0.2, 0.5}; for the sensitivity analysis in Fig. 3 we extended this to {0.01, 0.05, 0.1, 0.2, 0.5, 1.0}. Learning rates were tuned under cosine decay: for CIFAR we considered {0.01, 0.1}, with 0.1 consistently selected, and we considered {10^−2^, 10^−3^, 10^−4^} for BUS and COVID-19 due to smaller dataset size. Batch sizes were fixed at 128 for CIFAR and drawn from {8, 16, 64} for medical datasets to respect GPU memory constraints. For Adam-based methods, we tuned the weight decay parameter over {1 × 10^−4^, 5 × 10^−4^, 1 × 10^−3^}, while momentum parameters were fixed at $\beta_{1}=0.9$ and $\beta_{2}=0.999$.

#### 1) VALIDATION PROTOCOL

For CIFAR-10/100, we followed the standard dataset split (50k train / 10k test). During hyperparameter selection, we carved out 10% of the training set (45k train / 5k validation / 10k test). After selecting the best hyperparameters, the final models were trained once on the full 50k training set and evaluated on the 10k test set. For BUS and COVID-19, we first created a fixed 80%–20% stratified train–test split. Hyperparameters were then selected by stratified 5-fold cross-validation within the 80% training partition, and the final model was trained on the entire 80% training portion with the chosen hyperparameters and evaluated once on the held-out 20% test set.

#### 2) SELECTION RULE

Grid search was performed jointly across candidate values. Each candidate configuration was trained three times with different random seeds, and the mean validation accuracy was used for comparison. When two configurations produced statistically indistinguishable results (i.e., mean validation accuracies differing by less than one pooled standard deviation across seeds), we chose the simpler configuration (e.g., larger batch size or higher learning rate) to favor efficiency and reproducibility. Early stopping was applied uniformly to prevent overfitting. Final test results throughout the paper are reported as mean ± standard deviation over three independent runs.

This unified strategy ensures that all optimizers were tuned under the same protocol, and that the improvements of GCSAM reflect algorithmic advantages rather than hyperparameter bias.

### B. IMAGE CLASSIFICATION IN GENERAL DOMAIN

To evaluate the general effectiveness of GCSAM across multiple architectures and tasks, we conduct comparative experiments on both the CIFAR-10 and CIFAR-100 datasets [47]. CIFAR-10 contains 60000 images across 10 classes, while CIFAR-100 includes 100 classes with finer-grained visual categories. We compare GCSAM against several baselines: the Adam optimizer, Gradient Centralization (GC) applied to Adam, the original SAM, and four SAM variants (ASAM, GSAM, CRSAM, and MSAM). These methods are evaluated on four widely adopted deep learning architectures: ResNet-50 [43], VGG-16 [44], Vision Transformer (ViT) [45], and Swin Transformer [46]. For CIFAR-10 and CIFAR-100, we followed the unified hyperparameter tuning strategy described in Section IV-A. All models were trained from scratch with a fixed batch size of 128 and cosine learning rate decay [48]. Standard CIFAR augmentations were applied, including random crop with 4-pixel padding, horizontal flip, and normalization. Label smoothing [49] with a factor of 0.1 was used to improve generalization and calibration. Final results are reported as mean ± standard deviation.

We present the results of our experiments on CIFAR-10 and CIFAR-100 using Adam, GC, SAM, four SAM variants (ASAM, GSAM, CRSAM, MSAM), and our proposed GCSAM optimizer, as shown in Table 1. Across all architectures, Adam provides the lowest performance, while applying GC alone yields modest but consistent improvements, confirming its utility as a standalone technique. The original SAM improves further, and some SAM variants (e.g., ASAM, GSAM) provide occasional gains over SAM depending on the architecture, though the improvements are not consistent across all variants and datasets. Nevertheless, GCSAM consistently achieves the highest test accuracies on both CIFAR-10 and CIFAR-100. For example, on CIFAR-10, GCSAM reaches 96.24% with ResNet-50, 95.87% with VGG-16, 87.67% with ViT, and 91.12% with Swin Transformer, surpassing the best-performing SAM variant in each case. On CIFAR-100, GCSAM also leads, obtaining 82.03% with ResNet-50, 79.52% with VGG-16, 74.25% with ViT, and 75.76% with Swin Transformer. These results demonstrate that while SAM and some of its variants can provide improvements over Adam, integrating gradient centralization within the sharpness-aware framework yields consistently stronger and more stable gains, highlighting the effectiveness of GCSAM across both convolutional and transformer-based architectures.

To analyze robustness to the perturbation radius ρ, we evaluated SAM and GCSAM across 0.01, 0.05, 0.1, 0.2, 0.5, 1.0 on CIFAR-10 with four architectures (ResNet-50, VGG-16, ViT, Swin Transformer). Results in 3 show that SAM achieves its best accuracy around ρ=0.05 but exhibits sharp performance drops as ρ increases or decreases. In contrast, GCSAM achieves peak accuracy around ρ=0.1 while maintaining stable performance across the full range of ρ values, confirming that gradient centralization reduces sensitivity to this hyperparameter. The smaller variance of GCSAM across architectures indicates its robustness and practical advantage, as less fine-tuning of ρ is required.

### C. IMAGE CLASSIFICATION IN MEDICAL DOMAIN

Generalization is particularly challenging in the medical domain, where images are collected using a variety of devices, imaging protocols, and across diverse patient populations. These factors introduce unique complexities that impact model performance on unseen data. Prior studies have demonstrated that SAM consistently outperforms traditional optimizers like Adam and SGD across various models due to its ability to achieve flatter minima [50], [51]. To assess the efficacy of GCSAM in tackling these domain-specific challenges, we conduct extensive experiments using two medical datasets: breast ultrasound (BUS) images and COVID-19 chest X-ray images. These datasets are chosen to evaluate the algorithm’s robustness under varying imaging conditions and domain shifts. Both BUS and COVIDx datasets are publicly available, anonymized, and released under institutional ethical approvals, ensuring compliance with ethical standards for research using de-identified medical data.

#### 1) BREAST ULTRASOUND (BUS) DATASET

We constructed a comprehensive breast ultrasound dataset by combining 3,641 images from [52] with 2,405 images from the GDPH&SYSUCC dataset [53], resulting in a total of 6,046 images labeled as benign or malignant. To ensure fair and consistent evaluation, we applied an 80%–20% stratified train–test split, preserving the original class distribution. Hyperparameter tuning followed the unified protocol described in Section IV-A, with the learning rate sweep restricted to {10^−2^, 10^−3^, 10^−4^} and batch sizes to {8, 16, 64} due to dataset size and memory constraints. Final test accuracies are reported as mean ± standard deviation over three runs with different random seeds.

The results in Table 2 show that GCSAM consistently achieves the highest performance across all architectures on the BUS dataset. Applying GC alone yields modest gains over Adam, while the original SAM improves further. Some recent SAM variants (e.g., ASAM, GSAM) provide modest improvements over SAM in certain architectures, but the gains are not consistent across all variants and models. Nevertheless, GCSAM surpasses all baselines. For example, ResNet-50 improves from 77.14% with Adam and 78.65% with SAM to 80.07% with GCSAM. VGG-16 reaches the highest overall accuracy of 83.28% with GCSAM, compared to 82.71% with SAM and 82.87% with the best SAM variant (GSAM). For transformer-based models, which are generally more sensitive to optimization settings, GCSAM still yields clear improvements: ViT increases from 70.43% (Adam) and 71.10% (best variant, GSAM) to 72.18%, while Swin Transformer improves from 69.33% (Adam) and 70.21% (best variant, SAM) to 70.74%. These results highlight that while SAM variants can provide occasional accuracy gains, integrating gradient centralization within the sharpness-aware framework yields more consistent and robust improvements, even in challenging medical imaging tasks with relatively small datasets. The low standard deviations across all runs further confirm the stability and reproducibility of our approach.

#### 2) COVID-19 CHEST X-RAY DATASET

We conducted additional experiments on the COVIDx CXR-4 dataset [54], comprising 16,955 chest X-ray images categorized into COVID-positive and negative cases. To evaluate the generalization ability of our optimizer, we trained ResNet-50, VGG-16, Vision Transformer (ViT), and Swin Transformer models. We compared the performance of GCSAM against the Adam optimizer, SAM, and several notable SAM variants, including Adaptive SAM (ASAM) [8], Surrogate-Gap SAM (GSAM) [9], Curvature-Regularized SAM (CRSAM) [10], and Momentum SAM (MSAM) [28]. To quantify model sharpness, we employed the PyHessian framework [55] to compute the top eigenvalue of the Hessian matrix, providing a principled measure of the loss landscape’s curvature. This allowed us to analyze how well each optimizer controls sharpness and contributes to better generalization. In addition to accuracy and sharpness, we assessed the computational efficiency of GCSAM relative to SAM and its variants. Using Adam as a baseline with a normalized relative speed of 1.0, we benchmarked training times to evaluate any computational overhead introduced by GCSAM. This comprehensive evaluation highlights GCSAM’s effectiveness in improving both generalization and sharpness, while maintaining competitive training efficiency.

We followed the unified hyperparameter tuning strategy described in Section IV-A for the COVID-19 dataset. In line with the smaller dataset size, the learning rate sweep was restricted to {10^−2^, 10^−3^, 10^−4^} and batch sizes to {8, 16, 64}. All sharpness-aware optimizers were tuned over *ρ* ∈ {0.05, 0.1, 0.2, 0.5}, consistent with the global protocol. Final test accuracies are reported as mean ± standard deviation over three runs with different random seeds.

In Table 3, we report the test accuracy, computational cost, and top hessiane eigenvalue for each optimizer across four models: ResNet50, VGG16, ViT, and Swin Transformer, trained on the COVID-19 CXR-4 dataset. We observe that GCSAM consistently achieves higher test accuracy than the baseline Adam optimizer, SAM and its variants across all models, highlighting its effectiveness in improving generalization performance on medical image classification tasks. For ResNet50, GCSAM yields an accuracy of 90.81%, surpassing all other optimizers, with ASAM being the closest competitor at 90.21%. Similarly, for VGG16, GCSAM achieves 92.03%, which is higher than the next best, SAM at 91.43%. The trend continues with ViT, where GCSAM outperforms all optimizers with 82.52%, and on the Swin Transformer, it achieves 85.93%, surpassing SAM at 85.59% and MSAM at 83.87%. In terms of computational cost, GCSAM introduces a moderate overhead relative to Adam but generally exhibits better efficiency compared to SAM. To facilitate a clear comparison, we present the training speed of each optimizer relative to Adam, with Adam’s speed normalized to 1.0. This normalization allows for a direct evaluation of the computational impact of each optimizer. The results indicate that GCSAM strikes an optimal balance, maintaining competitive efficiency while achieving significant improvements in accuracy.

Additionally, the Hessian values provide crucial insights into the geometry of the loss landscape. A lower Hessian value is indicative of a flatter, more stable loss landscape, which is desirable for the optimization process as it suggests that the model is less likely to get trapped in sharp, narrow minima that could lead to poor generalization. GCSAM consistently demonstrates the lowest Hessian values across all models, with values of 164.32 for ResNet50 and 139.72 for VGG16, which are substantially lower than those of other optimizers. For comparison, GSAM and CRSAM exhibit much higher Hessian values, indicating sharper loss landscapes. These results suggest that GCSAM promotes a smoother and more stable optimization path, potentially leading to better generalization performance by encouraging the model to converge to flatter minima. This further underscores the advantages of GCSAM in terms of both accuracy and the optimization stability it provides across different neural network architectures on the COVID-19 dataset.

### D. ABLATION STUDY

#### 1) GC PLACEMENT

Sharpness-Aware Minimization (SAM) requires two gradient computations per update: an ascent gradient to form the perturbation ϵ, and a descent gradient to update the parameters. Gradient Centralization (GC) can, in principle, be applied to either or both of these gradients. To determine the optimal placement, we conducted an ablation study on ResNet-50 using the BUS and CIFAR-10 datasets, comparing five configurations: Adam, SAM without GC, GC applied only in the ascent step (our proposed GCSAM), GC applied only in the descent step, and GC applied in both ascent and descent steps.

As shown in Table 4, applying GC only in the ascent step achieves the highest performance on both datasets, reaching 80.07% on BUS and 96.24% on CIFAR-10. Descent-only GC provides minor improvements over Adam but fails to match SAM, while GC in both steps improves stability but yields no additional accuracy gains beyond ascent-only. These results indicate that the ascent step is the critical locus for applying GC to enhance generalization.

**FIGURE 4.**
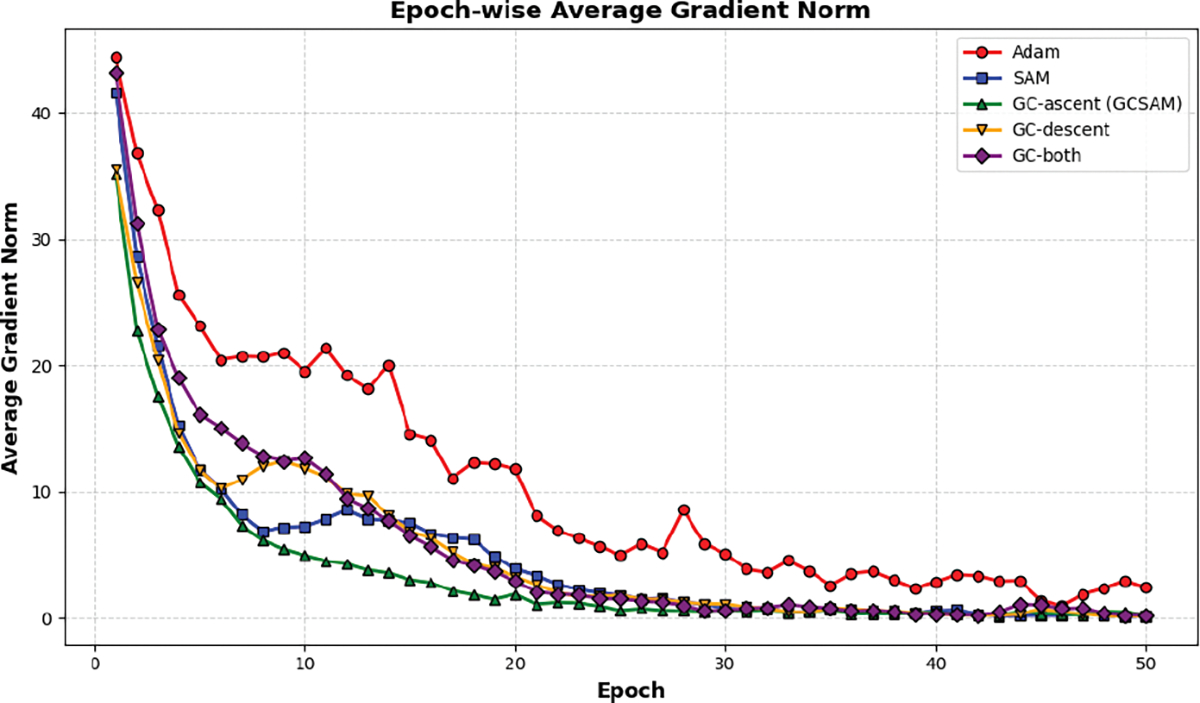
Epoch-wise average $\ell_{2}$ gradient norm on the BUS dataset (ResNet-50) for different GC placements. Ascent-only GC achieves the lowest and most stable norms, confirming its effectiveness in stabilizing the ascent step of SAM.

**FIGURE 5.**
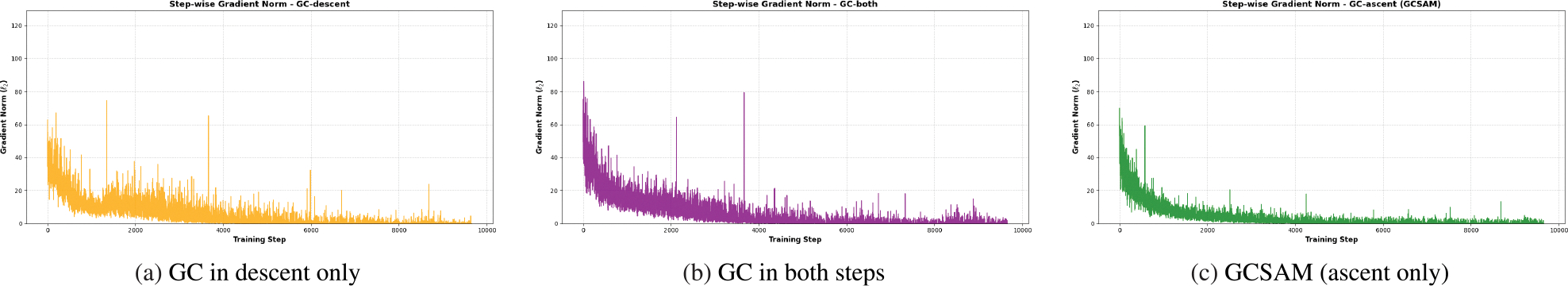
Step-wise $\ell_{2}$ gradient norm traces on the BUS dataset (ResNet-50) comparing different GC placements. Extending across the full page, this figure highlights that GCSAM (ascent-only) consistently achieves the lowest and most stable dynamics throughout training, while descent-only and both-step configurations remain less effective.

To further assess training stability, Fig. 4 shows the epoch-wise average $\ell_{2}$ gradient norm on BUS, while Fig. 5 provides detailed step-wise traces for the three GC placement strategies. Descent-only GC modestly reduces scale but leaves instability largely intact. Both-step GC smooths the trajectory but still produces higher variance than ascent-only. In contrast, ascent-only GC achieves the lowest and most stable gradient norms, eliminating early-stage spikes while preserving effective descent steps.

These findings align with our theoretical analysis in Appendix A. Instability in SAM arises when the ascent gradient used to define ϵ contains redundant or noisy components. Applying GC to the ascent step regularizes this perturbation, ensuring that the adversarial neighborhood is probed stably while leaving the descent update intact. By contrast, descent-only GC alters the update direction itself, which may suppress informative directions, and both-step GC combines these drawbacks. Consequently, ascent-only GC provides the best balance between stability and generalization, validating the core design of GCSAM.

#### 2) SCHEDULING

SAM is known to sometimes exhibit instability in the early stages of training, especially when combined with SGD. A common remedy is to warm-start with a vanilla optimizer before switching to SAM or its variants. To evaluate whether this improves GCSAM, we conducted an ablation on the BUS dataset using ResNet-50, comparing always-GCSAM against a 10-epoch warm-start with Adam followed by GCSAM or SAM. Results are reported in Table 5.

As shown in Table 5, warm-starting with Adam for the first 10 epochs yields only a marginal improvement over always-GCSAM, with overlapping standard deviations. Warm → SAM improves stability compared to always-SAM but still underperforms GCSAM-based methods. This suggests that ascent-based GC already suppresses early-stage perturbation noise, making explicit warm-start scheduling largely redundant.

#### 3) GC-EMA VARIANTS

We also evaluated a temporal smoothing variant of GC, denoted GC-EMA, which maintains an exponential moving average (EMA) of the gradient mean with momentum β. At each step t, GC-EMA updates

$$\mu_{t}=\beta \mu_{t-1}+(1-\beta )mean\left(g_{t}\right),$$

and subtracts $\mu_{t}$ from $g_{t}$ instead of the instantaneous mean. Results for β=0.9 and β=0.99 on BUS (ResNet-50) are reported in Table 6.

Table 6 shows that GC-EMA yields nearly identical test accuracy to per-step GC, with differences well within the margin of standard deviation. Although GC-EMA produces slightly smoother gradient-norm traces, it adds an additional hyperparameter and complicates interactions with optimizer moments. For clarity and efficiency, we adopt per-step GC as the default throughout the paper, noting that GC-EMA remains a potential variant for applications where temporal smoothing is specifically required.

Together, these ablations indicate that ascent-based per-step GC already mitigates early-stage instability, reducing the need for scheduling or optimizer switching, and per-step GC is a simpler and equally effective choice compared to GC-EMA. These findings further validate the design decisions underlying GCSAM.

## V. DISCUSSION

In this section, we analyze the results presented in Table 1, Table 2, and Table 3, highlighting the improved generalization and training efficiency offered by the GCSAM optimizer in comparison to SAM and the baseline Adam optimizer.

### A. BETTER GENERALIZATION PERFORMANCE

Across the CIFAR-10 and CIFAR-100 datasets (Table 1), GCSAM achieves higher test accuracies than both SAM and Adam on all four tested architectures: ResNet50, VGG16, ViT, and Swin Transformer. This demonstrates that GCSAM’s ability to mitigate gradient explosion in the ascent step of SAM results in more effective training and better model robustness on unseen data. Moreover, GCSAM’s generalization improvements are more pronounced in medical imaging tasks, where domain shifts and data variability pose significant challenges to traditional optimization methods. For instance, in breast ultrasound (BUS) images (Table 2) and COVID-19 chest X-rays (Table 3), GCSAM consistently outperforms SAM and Adam, demonstrating its resilience to the inherent variability of medical image datasets. Specifically, for the COVID-19 dataset, GCSAM surpasses all tested SAM variants, further establishing its effectiveness in challenging domains.

While GCSAM improves both CNNs and ViTs, the latter show smaller absolute gains in medical imaging tasks. This performance gap can be attributed to two main factors. First, ViTs typically require much larger training datasets to achieve optimal performance, whereas our medical datasets (BUS: 6,046 images, COVID-19: 16,955 images) are relatively small for transformer-based models and limit their ability to exploit global attention effectively. Second, CNNs possess stronger inductive biases for local feature extraction through convolutional operations, making them better suited for capturing the texture and edge-level information that is especially important in medical images. Nevertheless, GCSAM consistently improves the stability and generalization of ViTs compared to SAM and Adam, demonstrating its effectiveness even in data-limited regimes.

**FIGURE 6.**
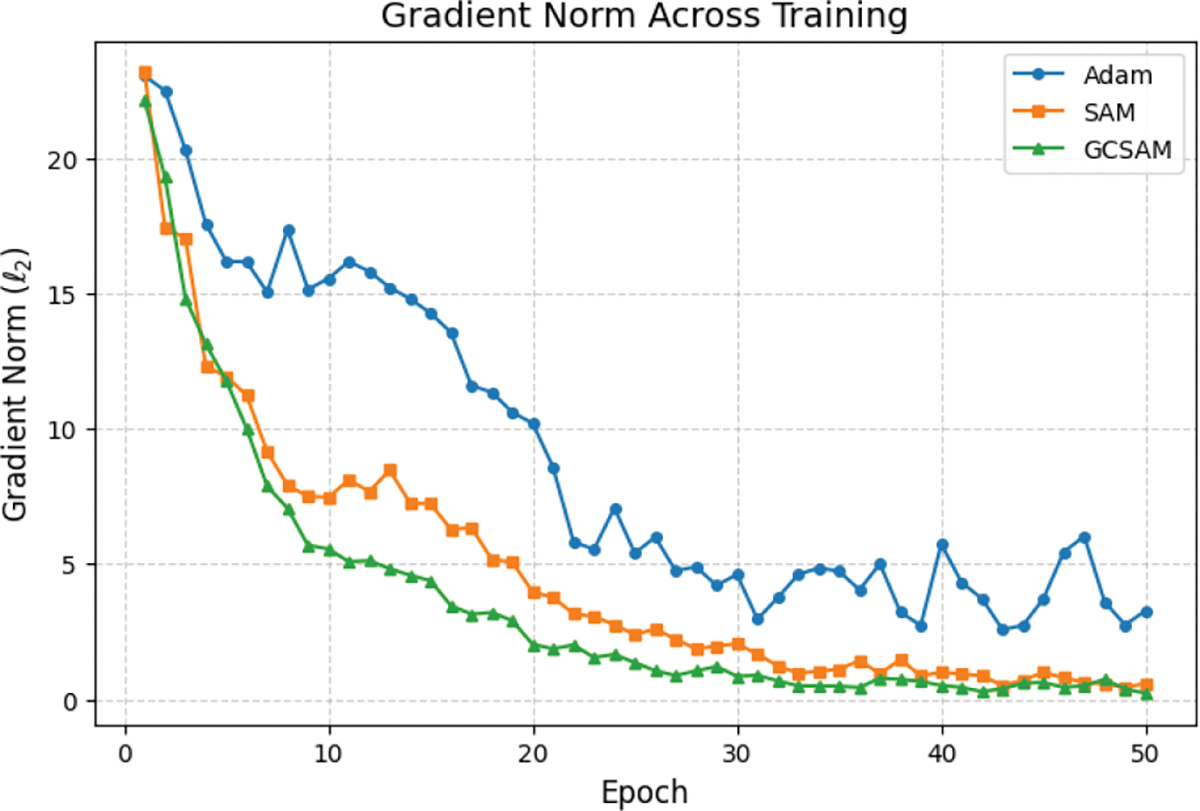
Average gradient norm per epoch for Adam, SAM, and GCSAM. GCSAM consistently maintains lower gradient norms, demonstrating its ability to suppress gradient explosion and stabilize training.

To enhance generalization across all tested models, GCSAM focuses on three core aspects: sharpness reduction, gradient centralization, and suppression of gradient explosion. GCSAM promotes convergence to flatter minima in the loss landscape, which are associated with better generalization and reduced sensitivity to input perturbations. It also applies gradient centralization by subtracting the mean of the gradient elements for each layer, which reduces redundancy in updates and encourages smoother optimization dynamics. Crucially, GCSAM mitigates the risk of gradient explosion, particularly during the ascent step of SAM where gradients are perturbed to explore sharp regions. By stabilizing these updates, GCSAM prevents the emergence of excessively large gradients and ensures more controlled parameter changes. This stabilizing effect is visually supported by the gradient norm trends in Fig. 6, where GCSAM consistently maintains lower gradient norms compared to both Adam and SAM throughout training. Together, these improvements result in better test accuracy and more robust generalization across both general-purpose and medical imaging datasets.

To provide a comprehensive view of optimization stability, we analyze the full trajectory of the gradient norm throughout training (i.e., over all iterations rather than epoch-wise averages). This allows us to directly observe instances of gradient explosion or instability across different optimizers.

**FIGURE 7.**
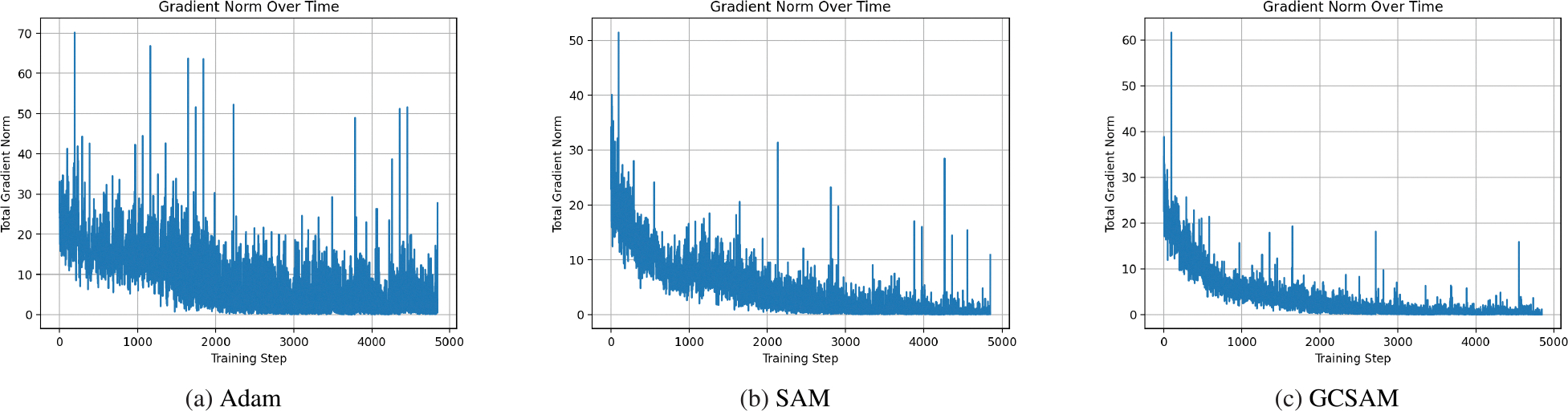
Step-wise $\ell_{2}$ gradient norm during training for Adam, SAM, and GCSAM on Covid-19 dataset. Adam and SAM exhibit unstable and occasionally exploding gradients, while GCSAM maintains consistently lower and smoother gradients throughout training, indicating improved optimization stability.

Fig. 7 illustrates the $\ell_{2}$ norm of the gradients computed at each training step for Adam, SAM, and the proposed GCSAM. It is evident that both Adam and SAM exhibit significantly higher fluctuations and, in some cases, signs of gradient explosion, especially in the early or mid-training stages. In contrast, GCSAM maintains a more stable gradient magnitude throughout the optimization process, indicating improved robustness and smoother convergence.

These results further support our claim that incorporating gradient centralization into sharpness-aware optimization mitigates instability and prevents the optimizer from entering regions with extreme curvature or sharp loss landscapes.

### B. BETTER COMPUTATIONAL EFFICIENCY

GCSAM provides computational advantages over SAM, as demonstrated in Table 3, by achieving lower relative training times across all models. By suppressing gradient spikes, GCSAM promotes faster convergence and reduces the number of required iterations. This is especially beneficial for large-scale models and datasets, where SAM’s ascent step incurs significant overhead. Additionally, GCSAM’s use of gradient centralization stabilizes updates, further lowering computational costs by guiding optimization toward stable regions of the loss landscape. Overall, GCSAM achieves an effective balance between generalization and efficiency.

### C. VISUALIZATION OF LOSS LANDSCAPES

**FIGURE 8.**
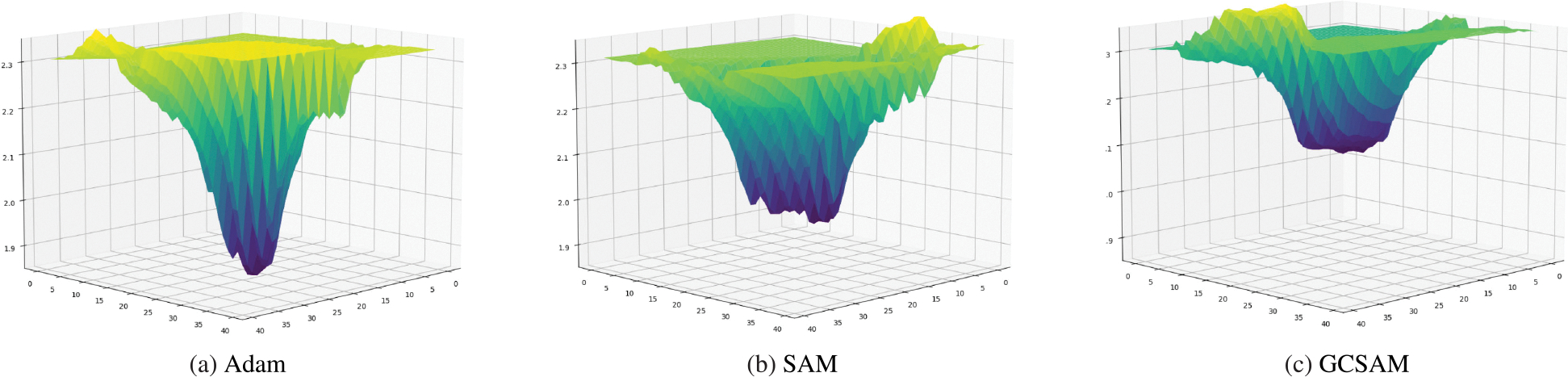
Comparison of loss landscapes for Adam, SAM, and GCSAM on the COVID-19 dataset. Adam demonstrates the sharpest minima, reflecting less stable convergence. In contrast, GCSAM achieves the flattest minima, indicative of improved stability and better convergence properties.

To analyze the geometry of the loss surface achieved by GCSAM, we visualized the loss landscapes of ResNet50 models trained with Adam, SAM, and GCSAM on the COVID-19 dataset. Following the methodology in [6], loss values were computed along two orthogonal Gaussian perturbation directions centered at the final trained weights. As shown in Fig. 8, GCSAM leads to consistently flatter minima than both Adam and SAM. This visual flatness is consistent with the lower dominant Hessian eigenvalues observed in our sharpness analysis, further supporting GCSAM’s ability to guide optimization toward flatter and more generalizable solutions.

### D. ABLATION INSIGHTS: GC PLACEMENT, SCHEDULING, AND EMA

Our ablation studies further clarify the design choices underlying GCSAM. First, we evaluated different placements of gradient centralization and found that applying GC only to the ascent step yields the best generalization performance. This configuration stabilizes the perturbation direction in SAM without altering the descent update itself, striking an optimal balance between stability and accuracy. By contrast, applying GC in the descent step or in both steps produced either reduced test accuracy or negligible additional gains, despite sometimes yielding smoother gradient-norm traces. Second, we examined warm-start scheduling (switching from a vanilla optimizer to GCSAM after a few epochs) and observed only marginal improvements over always-GCSAM, indicating that ascent-based GC already mitigates early-stage instability. Finally, we tested an EMA variant of GC (GC-EMA), which maintains a running mean of gradients rather than per-step subtraction. While GC-EMA slightly smooths gradient trajectories, it provided no meaningful accuracy improvements and introduced an additional hyperparameter. Together, these findings reinforce that the core design of GCSAM offers the best trade-off between simplicity, stability, and generalization, without requiring additional scheduling or smoothing mechanisms.

### E. LIMITATIONS

Although GCSAM consistently improves generalization and training stability across diverse architectures and datasets, several practical considerations remain. Like other sharpness-aware optimizers, GCSAM requires two backward passes per iteration, which increases training cost compared to single-pass optimizers. Future work could explore incorporating momentum-based strategies within GCSAM to further reduce computational costs, making it more efficient for large-scale applications. In addition, while GCSAM reduces sensitivity to the perturbation radius ρ, adaptive strategies for automatic hyperparameter tuning could further enhance its usability across diverse problem domains. Finally, extending GCSAM beyond vision tasks to natural language processing and multimodal learning represents a promising direction for broadening its impact.

## VI. CONCLUSION

In this work, we introduced Gradient-Centralized Sharpness-Aware Minimization (GCSAM), a novel optimizer designed to enhance model generalization and training stability. Across both standard vision benchmarks (CIFAR-10, CIFAR-100) and challenging medical imaging datasets (breast ultrasound and COVID-19 chest X-rays), GCSAM consistently outperformed Adam and SAM in terms of test accuracy and convergence behavior. By stabilizing gradients during the ascent step, GCSAM reduces variance, accelerates convergence, and alleviates the sensitivity of SAM to the perturbation radius, thereby simplifying practical deployment. These properties make GCSAM particularly valuable in domains where robust generalization is essential, such as medical imaging, where domain shifts and acquisition variability are common. Looking ahead, GCSAM provides a foundation for developing more efficient sharpness-aware methods, with opportunities to integrate momentum-based updates, adaptive hyperparameter tuning, and extensions to non-vision modalities.

## Figures and Tables

**TABLE 1.** Mean test accuracy (%) with standard deviation on CIFAR-10 and CIFAR-100. Results are averaged over three runs.

Model / Dataset	Adam	GC	SAM	ASAM	GSAM	CRSAM	MSAM	GCSAM

ResNet-50 / CIFAR-10	95.19 ± 0.16	95.46 ± 0.14	95.82 ± 0.18	95.89 ± 0.16	95.74 ± 0.15	95.55 ± 0.15	95.62 ± 0.14	**96.24** ± 0.13
ResNet-50 / CIFAR-100	80.32 ± 0.25	80.67 ± 0.21	81.15 ± 0.22	81.28 ± 0.20	81.23 ± 0.19	81.16 ± 0.18	81.21 ± 0.19	**82.03** ± 0.19

VGG-16 / CIFAR-10	94.84 ± 0.12	95.07 ± 0.15	95.31 ± 0.12	95.41 ± 0.14	95.47 ± 0.13	95.40 ± 0.13	95.34 ± 0.13	**95.87** ± 0.19
VGG-16 / CIFAR-100	77.89 ± 0.31	78.21 ± 0.28	78.95 ± 0.28	79.03 ± 0.26	79.10 ± 0.25	79.02 ± 0.24	78.85 ± 0.25	**79.52** ± 0.24

ViT / CIFAR-10	86.29 ± 0.21	86.55 ± 0.18	87.39 ± 0.16	87.27 ± 0.15	87.30 ± 0.14	87.13 ± 0.14	87.29 ± 0.15	**87.67** ± 0.11
ViT / CIFAR-100	72.17 ± 0.35	72.49 ± 0.31	73.42 ± 0.30	73.11 ± 0.29	73.26 ± 0.28	73.19 ± 0.27	73.04 ± 0.28	**74.25** ± 0.27

Swin / CIFAR-10	89.03 ± 0.28	89.35 ± 0.23	90.61 ± 0.25	90.14 ± 0.23	90.08 ± 0.22	90.52 ± 0.21	89.78 ± 0.22	**91.12** ± 0.21
Swin / CIFAR-100	73.05 ± 0.33	73.44 ± 0.30	74.88 ± 0.29	74.01 ± 0.28	74.09 ± 0.27	74.72 ± 0.26	73.75 ± 0.27	**75.76** ± 0.25

**TABLE 2.** Mean test accuracy (%) with standard deviation on the BUS dataset using Adam, GC, SAM, four SAM variants, and GCSAM. Results are averaged over three runs.

Model	Adam	GC	SAM	ASAM	GSAM	CRSAM	MSAM	GCSAM

ResNet-50	77.14 ± 0.25	77.52 ± 0.23	78.65 ± 0.18	78.92 ± 0.19	77.97 ± 0.18	78.01 ± 0.18	78.75 ± 0.19	**80.07** ± 0.15
VGG-16	81.69 ± 0.22	82.01 ± 0.21	82.71 ± 0.27	82.72 ± 0.25	82.87 ± 0.24	82.02 ± 0.23	81.96 ± 0.24	**83.28** ± 0.23
ViT	70.43 ± 0.35	70.51 ± 0.32	70.29 ± 0.31	70.85 ± 0.30	71.10 ± 0.29	70.94 ± 0.29	70.89 ± 0.30	**72.18** ± 0.25
Swin Transformer	69.33 ± 0.28	69.62 ± 0.26	70.21 ± 0.24	70.15 ± 0.23	70.02 ± 0.23	69.55 ± 0.22	69.68 ± 0.23	**70.74** ± 0.20

**TABLE 3.** Generalization performance (Test Accuracy %), computational cost (Speed), and top Hessian eigenvalue (Hessian) for Adam, SAM, Adaptive SAM (ASAM), Surrogate-Gap SAM (GSAM), Curvature-Regularized SAM (CRSAM), Momentum SAM (MSAM), and Gradient-Centralized SAM (GCSAM) on COVID-19 dataset.

Optimizer	ResNet50			VGG16		
	Test Accuracy	Speed	Hessian	Test Accuracy	Speed	Hessian

Adam	86.13±0.25	1.00	28523.72	90.71±0.16	1.00	1274.21
SAM	89.25±0.19	2.27	1384.16	91.43±0.24	1.89	274.13
ASAM	90.21±0.26	3.45	953.21	91.08±0.27	3.83	387.19
GSAM	88.48±0.29	4.91	3792.17	91.52±0.31	4.67	874.49
CRSAM	86.69±0.22	1.98	9537.24	90.82±0.16	1.73	973.15
MSAM	87.35±0.11	**1.14**	1952.42	90.32±0.24	**1.09**	935.61
GCSAM	**90.81**±0.21	1.93	**164.32**	**92.03**±0.19	1.83	**139.72**

	ViT			Swin Transformer		
Optimizer	Test Accuracy	Speed	Hessian	Test Accuracy	Speed	Hessian

Adam	81.71±0.19	1.00	846.13	84.62±0.29	1.00	2549.17
SAM	82.21±0.11	1.93	163.49	85.59±0.24	2.03	549.61
ASAM	81.56±0.24	3.85	96.21	82.63±0.35	3.41	2374.46
GSAM	80.93±0.30	4.79	890.38	83.18±0.21	4.57	1654.71
CRSAM	80.59±0.18	1.84	896.14	84.06±0.28	1.89	2491.65
MSAM	81.42±0.32	**1.12**	952.17	83.87±0.26	**1.19**	1735.73
GCSAM	**82.52**±0.18	1.85	**132.69**	**85.93**±0.21	1.87	**117.62**

**TABLE 4.** Ablation study of GC placement on BUS and CIFAR-10 datasets (ResNet-50). Results are mean test accuracy (% ± std) averaged over three runs. Baseline Adam, SAM, and GCSAM (ascent-only) values are taken from Tables 1 and 2.

Method	BUS	CIFAR-10

Adam	7.14 ± 0.20	95.19 ± 0.16
SAM (no GC)	8.65 ± 0.18	95.82 ± 0.18
GCSAM (ascent only)	**80.07 ± 0.15**	**96.24 ± 0.13**
GC in descent only	7.92 ± 0.19	95.34 ± 0.17
GC in both ascent & descent	8.98 ± 0.16	96.03 ± 0.19

**TABLE 5.** Scheduling ablation on BUS (ResNet-50). Results are mean test accuracy (% ± std) over three runs. “Warm → X” denotes Adam for 10 epochs then switching to X.

Method	BUS	CIFAR-10

GCSAM (always)	80.07 ± 0.15	96.24 ± 0.13
Warm → GCSAM	**80.19 ± 0.15**	**96.31 ± 0.19**
SAM (always)	78.65 ± 0.18	95.82 ± 0.18
Warm → SAM	78.95 ± 0.21	95.93 ± 0.16

**TABLE 6.** GC-EMA ablation on BUS (ResNet-50). Results are mean test accuracy (% ± std) over three runs.

Method	BUS	CIFAR-10

GCSAM (per-step GC)	80.07 ± 0.15	96.24 ± 0.13
GCSAM + GC-EMA (*β* = 0.9)	**80.12 ± 0.19**	**96.29 ± 0.15**
GCSAM + GC-EMA (*β* = 0.99)	80.09 ± 0.17	96.26 ± 0.18

## APPENDIX A GENERALIZATION BOUND FOR GCSAM

Proof of Theorem 1:

### Proof:

Following the proof in SAM by Foret et al. [7], under the assumption that Gaussian perturbations in weight space do not improve the test error, the following PAC-Bayesian generalization bound holds:

(A.1)
$$\begin{array}{l} E_{\epsilon_{i}\sim 𝒩\left(0,\sigma^{2}\right)}\left[L_{D}(w+\epsilon )\right]\leq E_{\epsilon_{i}\sim 𝒩\left(0,\sigma^{2}\right)}\left[L_{S}(w+\epsilon )\right]\\ \;+\sqrt{\frac{1}{n\;-\;1}\left(k\;log\left(1+\frac{\|w{\|}_{2}^{2}}{\eta^{2}\rho^{2}}{\left(1+\sqrt{\frac{log\;n}{k}}\right)}^{2}\right)+4\;log\frac{n}{\delta }+O(1)\right)}. \end{array}$$

This can be upper bounded by

(A.2)
$$\begin{array}{l} L_{D}(w)\leq \;\underset{\|\epsilon {\|}_{p}\leq \rho }{max}\;L_{S}(w+\epsilon )\\ \;+\sqrt{\frac{1}{n\;-\;1}\left(k\;log\left(1+\frac{\|w{\|}_{2}^{2}}{\eta^{2}\rho^{2}}{\left(1+\sqrt{\frac{log\;n}{k}}\right)}^{2}\right)+4\;log\frac{n}{\delta }+O(1)\right)}. \end{array}$$

#### Step 1: Centralized gradient norm.

Using the definition of GC from Eq. (4)–(5), the centralized gradient is

(A.3)
$$\nabla_{GC}L_{S}(w)=P\nabla L_{S}(w),\;P=I-{ee}^{⊤}.$$

Its squared norm is

(A.4)
$${\left\|\nabla_{GC}L_{S}(w)\right\|}_{2}^{2}={\left\|\nabla L_{S}(w)\right\|}_{2}^{2}-{\left\|e^{⊤}\nabla L_{S}(w)\right\|}_{2}^{2}.$$

Since ${\left\|e^{⊤}\nabla L_{S}(w)\right\|}_{2}^{2}\geq 0$, we obtain the inequality

(A.5)
$${\left\|\nabla_{GC}L_{S}(w)\right\|}_{2}^{2}\leq {\left\|\nabla L_{S}(w)\right\|}_{2}^{2}.$$

#### Step 2: Implication for sharpness bound.

Inequality (A.5) shows that the centralized gradient has strictly smaller (or equal) norm than the original gradient. Consequently, the adversarial perturbation in GCSAM,

$$\epsilon_{GC}=\rho \cdot \frac{\nabla_{GC}L_{S}(w)}{{\left\|\nabla_{GC}L_{S}(w)\right\|}_{2}},$$

induces a smaller or equal worst-case loss than the corresponding SAM perturbation.

#### Step 3: Tighter bound.

Therefore, the PAC-Bayes bound for GCSAM becomes

(A.6)
$$\begin{array}{l} L_{D}(w)\leq \;\underset{{\left\|\epsilon_{GC}\right\|}_{p}\leq \rho }{max}\;L_{S}\left(w+\epsilon_{GC}\right)\\ \;+\sqrt{\frac{1}{n\;-\;1}\left(k\;log\left(1+\frac{\|w{\|}_{2}^{2}}{\eta^{2}\rho^{2}}{\left(1+\sqrt{\frac{log\;n}{k}}\right)}^{2}\right)+4\;log\frac{n}{\delta }+O(1)\right)}. \end{array}$$

Compared to the original SAM bound, which depends on the unnormalized gradient, this bound leverages the centralized gradient norm from Eq. (A.5). Because gradient centralization removes redundant gradient components, the resulting bound is strictly tighter, reducing the worst-case generalization gap and explaining the improved stability observed in practice. ▀
